# Transcriptomic analysis of the symbiotic responsivity trait in pea (Pisum sativum L.)

**DOI:** 10.18699/vjgb-25-28

**Published:** 2025-04

**Authors:** D.O. Kuzmina, E.A. Zorin, A.S. Sulima, D.A. Romanyuk, M.L. Gordon, A.I. Zhernakov, O.A. Kulaeva, G.A. Akhtemova, O.Y. Shtark, I.A. Tikhonovich, V.A. Zhukov

**Affiliations:** All-Russia Research Institute for Agricultural Microbiology, Pushkin 8, St. Petersburg, Russia; All-Russia Research Institute for Agricultural Microbiology, Pushkin 8, St. Petersburg, Russia; All-Russia Research Institute for Agricultural Microbiology, Pushkin 8, St. Petersburg, Russia; All-Russia Research Institute for Agricultural Microbiology, Pushkin 8, St. Petersburg, Russia; All-Russia Research Institute for Agricultural Microbiology, Pushkin 8, St. Petersburg, Russia; All-Russia Research Institute for Agricultural Microbiology, Pushkin 8, St. Petersburg, Russia; All-Russia Research Institute for Agricultural Microbiology, Pushkin 8, St. Petersburg, Russia; All-Russia Research Institute for Agricultural Microbiology, Pushkin 8, St. Petersburg, Russia; All-Russia Research Institute for Agricultural Microbiology, Pushkin 8, St. Petersburg, Russia; All-Russia Research Institute for Agricultural Microbiology, Pushkin 8, St. Petersburg, Russia; All-Russia Research Institute for Agricultural Microbiology, Pushkin 8, St. Petersburg, Russia

**Keywords:** pea, legume-rhizobial symbiosis, arbuscular mycorrhiza, symbiotic responsivity, transcriptomics, горох, бобово-ризобиальный симбиоз, арбускулярная микориза, симбиотическая отзывчивость, транскриптомика

## Abstract

Pea (Pisum sativum L.) is an important crop culture and a model object for studying the molecular genetic bases of nitrogen-fixing symbiosis and arbuscular mycorrhiza (AM). Pea genotypes with high and low responsivity to inoculation with nodule bacteria (rhizobia) and AM fungi have been described: the ‘responsive’ genotypes demonstrate an increase in seed weight under inoculation, while ‘non-responsive’ ones do not show such a reaction. In order to get insight into the molecular genetic mechanisms underlying the symbiotic responsivity, a transcriptomic analysis of whole root systems of pea plants of the ‘responsive’ genotype k-8274 (cv. Vendevil, France) and ‘non-responsive’ genotype k-3358 (unnamed cultivar, Saratov region, Russia) grown in soil without inoculation (control) and inoculated either with rhizobia (single inoculation) or with rhizobia together with AM fungi (double inoculation) was performed. It was shown that the ‘responsive’ genotype, indeed, demonstrated a pronounced transcriptomic response to single and double inoculation, in contrast to the ‘non-responsive’ genotype. In k-8274, single inoculation led to specific up-regulation of genes related to catabolism of polyamines, lipid metabolism, and jasmonic acid and salicylic acid signaling. Under double inoculation, the specifically up-regulated genes in k-8274 were related to arbuscular mycorrhiza infection, and the down-regulated genes were related to nodulation. This fact matches the phenotype of the plants: the number of nodules was lower in k-8274 under double inoculation as compared to the control. Thus, strict control over the nodule number may be one of the mechanisms underlying the symbiotic responsivity of pea. Finally, a comparison of expression profiles in k-8274 and k-3358 roots under double inoculation also allowed us to identify the transcriptomic signatures characteristic of the symbiotically responsive genotype. Further work will be focused on validation of these transcriptomic markers of the symbiotic responsivity trait in pea.

## Abstract (ru)

Горох посевной (Pisum sativum L.) является важной сельскохозяйственной культурой и модельным объектом для изучения молекулярно-генетических основ азотфиксирующего симбиоза и арбускулярной микоризы (АМ). Описаны генотипы гороха с высокой и низкой отзывчивостью на инокуляцию клубеньковыми бактериями (ризобиями) и АМ грибами: «отзывчивые» генотипы демонстрируют прибавку массы семян при инокуляции, а для «неотзывчивых» генотипов такая реакция не характерна. С целью описания молекулярно-генетических механизмов, лежащих в основе симбиотической отзывчивости, был проведен транскриптомный анализ целых корневых систем растений гороха «отзывчивого» генотипа к-8274 (сорт Vendevil, Франция) и «неотзывчивого» генотипа к-3358 (сорт из Саратовской области, Россия), выращенных в почве без инокуляции (контроль) и при инокуляции ризобиями (одиночная инокуляция) и ризобиями совместно с АМ грибами (двойная инокуляция). «Отзывчивый» генотип действительно продемонстрировал выраженный ответ на уровне транскриптома на одиночную и двойную инокуляцию, в отличие от «неотзывчивого» генотипа. Одиночная инокуляция привела у к-8274 к специфическому повышению экспрессии генов, связанных с катаболизмом полиаминов, метаболизмом липидов и сигналингом на основе жасмоновой и салициловой кислот. При двойной инокуляции у к-8274 была повышена экспрессия генов, связанных с арбускулярно-микоризной инфекцией, и понижена экспрессия генов, связанных с клубенькообразованием. Данный факт соответствует фенотипу растений: число клубеньков у к-8274 было снижено при двойной инокуляции по сравнению с контролем. Таким образом, одним из механизмов, лежащих в основе симбиотической отзывчивости у гороха, может быть строгий контроль над числом клубеньков. Наконец, сравнение экспрессионных профилей корней растений к-8274 и к-3358 в условиях двойной инокуляции позволило идентифицировать «транскриптомные сигнатуры», характерные для симбиотически отзывчивого генотипа к-8274. Дальнейшая работа будет направлена на подтверждение возможности использования выявленных генов в качестве транскриптомных маркеров признака симбиотической отзывчивости у гороха посевного.

## Introduction

Legume plants (family Fabaceae) are an important component of modern agricultural practices due to their ability to fix atmospheric nitrogen in symbiosis with nodule bacteria (rhizobia) (Rubiales, Mikic, 2015). The symbiotic nitrogen fixation provides plants with combined nitrogen, and this feature allows legumes to grow on lower doses of mineral fertilizers, which is economically advantageous and beneficial for the environment (Goyal et al., 2021). The N2 fixation occurs in specific organs called root nodules, which are formed on the roots of legume plants, where rhizobia are hosted within the plant cells (Yang et al., 2022). In addition to nitrogen fixation, legumes, like most terrestrial plants, form arbuscular mycorrhiza (AM), which helps plants cope with water deficiency and lack of mineral phosphorus (Smith, Read, 2008). This tendency to form mutualistic symbioses with beneficial microorganisms makes legumes profitable crops for use according to the concept of sustainable agriculture.

It is generally accepted that the root nodule (RN) symbiosis of legumes has evolved on the base of pre-existing AM (Parniske, 2008; Oldroyd, 2013). This idea is supported by the fact that some components of signaling systems are shared between the two symbioses. The first example of such shared signaling system is the so-called CSSP (common symbiosis signaling pathway), the signal transduction pathway that is activated during the early steps of the development of both AM and RN symbioses (Harrison, 2012; Wang D. et al., 2022). The second one is the autoregulation system, which exercises systemic control over the nodule number and the rate of mycorrhizal colonization of roots (autoregulation of nodulation (AON)/ autoregulation of mycorrhization (AOM)), depending on the amount of available nitrogen and phosphorus in the growth substrate, respectively (Reid et al., 2011; Ferguson et al., 2019; Müller, Harrison, 2019). Interestingly, nodulation systemically influences mycorrhization, and vice versa, as was shown in split-root experiments in alfalfa (Medicago sativa L.) (Catford et al., 2003).

Pea (Pisum sativum L.) is a profitable legume crop that has also served as a model to study the genetic system controlling the development of RN symbiosis and AM, similar to model legumes Medicago truncatula Gaertn., Lotus japonicus (Regel.) K. Larsen and Glycine max (L.) Merr. (Roy et al., 2020; Tsyganov, Tsyganova, 2020). Different genotypes have been described in pea with high and low responsivity to inoculation with rhizobia and AM fungi: the ‘responsive’ genotypes increase their seed productivity under inoculation, and the ‘non-responsive’ ones do not demonstrate such a reaction to inoculation (Shtark et al., 2006). This symbiotic responsivity trait was also called EIBSM, standing for Effectiveness of Interaction with Beneficial Soil Microorganisms, and was proposed for breeding of pea and other legumes (Shtark et al., 2012, 2015).

Recent development of pea genomics makes it possible to use post-genomic technologies such as transcriptomics and proteomics for studying agriculturally important traits (Parihar et al., 2022; Rubiales et al., 2023). Most such works, however, are devoted to studying resistance to pathogens rather than responsivity to symbionts (Castillejo et al., 2015; Cerna et al., 2017; Liu et al., 2023; Kälin et al., 2024), so the molecular mechanisms underlying the symbiotic responsivity in pea remain largely unexplored (Zhukov et al., 2021a). Therefore, the aim of this work was to reveal the molecular genetic bases of this trait by describing the transcriptomic changes in roots of two contrasting pea genotypes, the ‘responsive’ k-8274 and the ‘non-responsive’ k-3358, after inoculation with rhizobia (Rh) and rhizobia plus AM fungi (Rh+AM), as compared to a non-treated control

## Materials and methods

Vegetation experiments. The plant material (whole root systems) for RNA extraction was taken from the previously conducted vegetation experiment described in detail in (Zhukov et al., 2017). Briefly, the plants of the genotypes k-8274 and k-3358 (VIR collection of pea, St. Petersburg, Russia) were grown in non-sterile sod-podzolic light loamy soil (Leningrad Oblast, area of the Belogorka Science and Production Association, Chumus 1.27 % and Ntotal 0.11 %, pHsalt 4.92), three plants per 5-liter pot, in a greenhouse during the summer period at ARRIAM, St. Petersburg, Russia in the following variants: non-inoculated plants (control), plants inoculated with Rhizobium leguminosarum bv. viciae strain RCAM1026 (Rh), plants inoculated with Rhizobium leguminosarum bv. viciae strain RCAM1026 together with a mixture of arbuscular mycorrhizal fungi Rhizophagus irregularis BEG144, R. irregularis BEG53 and Glomus sp. ST3 (Rh+AM). After 4 weeks of growth, the plants were harvested; root systems were washed with water, immediately frozen in liquid nitrogen and stored at –80 °C until further processing. For a biological replicate, three plants from one pot were collected. In total, there were three biological replicates per treatment per plant genotype.

In the experiment on phenotypic characterization of k-8274 and k-3358, plants were grown in 2-liter pots with sterile sand, 4 plants per pot, without inoculation (control) and under inoculation with either Rhizobium leguminosarum bv. viciae RCAM1026 (Rh) or Rhizophagus irregularis BEG144 (AM), or their combination (Rh+AM), in four replicates (pots) per treatment. The seeds were pre-sterilized with concentrated sulphuric acid, rinsed 5 times with autoclaved distilled water, and germinated in Petri dishes on filter paper at 28 °C in the dark. Inoculation with rhizobia was performed by pouring 3 ml of water suspension (106 CFU×l–1) of Rh. leguminosarum bv. viciae RCAM1026 under each seedling; inoculation with AM fungi was performed by adding 60 g of dry roots of Sorghum sp. colonized by AM fungi (the details of the AM inoculation method are described in Shtark et al., 2019). At planting, the seedlings were supplied with mineral nutrition solution with a low content of phosphorus and nitrogen (Sulima et al., 2019), 150 ml per pot.

The plants were grown in a VB 1014 (Vötsch Industrietechnik, Germany) growth chamber under the following conditions: day/night 16/8 h, temperature 21 ± 1 °C, relative humidity 75 %, light irradiation 600 μmol of photons × m–2 × s–1. The plants were watered by 200 ml of distilled water twice a week. At 4 weeks after planting/inoculation, the plants were harvested, and the root systems were washed with water. The number of nodules was counted during visual examination of the root systems; the shoots were air-dried and weighted.

Statistical analysis was carried out in the R environment using the core package ‘stats’ (version 4.3.0). A two-way ANOVA was used to assess the effects of inoculations and their action on shoot dry weight, and a one-way ANOVA was used to evaluate the impact of mycorrhiza on nodulation.

RNA extraction, library preparation and sequencing. The frozen whole root systems were grinded in liquid nitrogen using mortars and pistils; the resulting powder was used for total RNA extraction using Trizol reagent (Thermo Fisher Scientific, USA). The quality of RNA was assessed using a 2100 Bioanalyzer Instrument (Agilent Technologies, USA). RNA of sufficient quality (RNA integrity number (RIN) > 8) was obtained from only two replicates of the samples from the control conditions, single inoculation and combined (double) inoculation, which allowed us to study the effect of single and combined inoculation on plants of the k-8274 and k-3358 genotypes.

The extracted RNA was used for RNAseq library preparation using the MACE v1.0 kit (GenXPro GmbH, Frankfurt am Main, Germany). The libraries were sequenced in GenXPro GmbH (Frankfurt am Main, Germany) on Illumina HiSeq2000. Raw sequencing data were uploaded to the NCBI database (BioProject number PRJNA1154300).

Bioinformatic analysis. The quality of raw reads was assessed using FastQC (version 0.11.9) (Andrews, 2010) and multiqc (Ewels et al., 2016). Trimmomatic (version 0.39) with default parameters was employed to remove adapter sequences and low-quality sequences (Bolger et al., 2014). Clean reads were aligned to the reference genome of cv. Frisson (NCBI: JANEYU000000000; Zorin et al., 2022) with STAR (version 2.7.10b) (Dobin et al., 2013) and sorted using SAMtools (version 1.17) (Danecek et al., 2021). Using multi-mapping, featureCounts (version 2.0.3) was used to count the number of reads that were aligned to genes or exons (Liao et al., 2014).

The BLAST+ command line tool (version 2.9.0) was used to annotate genes to which reads were mapped, with an E-value threshold of 1e–5 against Medicago truncatula functional annotation (genomic assembly MedtrA17_4.0) (Camacho et al., 2009). PCA plots were generated using the R packages DESeq2 and ggplot2 (Love et al., 2014; Wickham, 2016). R (version 4.1.3) was used to perform differential gene expression analysis with the DESeq2 package. Genes were considered differentially expressed if the Wald test was passed with the False Discovery Rate (FDR) value of no more than 0.05 and a log2-fold change less than or more than 0.5. Additionally, a targeted analysis of differential gene expression was performed on gene lists (listed in the Supplementary Table S1)1 that were acquired from earlier research projects. These gene lists included Sym genes and genes involved in the systemic process of autoregulation of nodulation.


Supplementary Materials are available in the online version of the paper: https://vavilovj-icg.ru/download/pict-2025-29/appx9.pdf


GO enrichment analysis was carried out using the topGO packages (version 2.42.0) (Alexa, Rahnenfuhrer, 2024), with the use of the weight01 method and Fisher’s exact test. Genes, the expression of which was considerably elevated or decreased, were used independently to search for biological processes. Biological pathways with statistically significant up/down-regulation were counted at p-value <0.05 and depicted using ggplot2. The p-value indicates the probability of this value occurring by chance, and suggests that the biological process under investigation is enriched in the transcriptomic data.

The heatmap is based on a matrix containing the values of 1–R (R is the Pearson correlation coefficient). The correlation is calculated based on the values of normalized expression (the number of normalized reads per million was obtained using DESeq2, VST (Variance Stabilizing Transformation)), which were additionally logarithmically transformed (log2), and then transformed into a z-scale, which for each gene for each sample reflects the number of standard deviations from the average value for all samples for this gene. Further, the genes were clustered based on these values using the hierarchical clustering method. The obtained matrix was displayed as heat maps with pheatmap (version 1.0.12) (Kolde, 2015). The R packages VennDiagram (version 1.7.3) and EnhancedVolcano (version 1.18.0) were used to show the results of the differential gene expression study (Chen, Boutros, 2011).

## Results

Transcriptomic response to single inoculation (Rh)

**Fig. 1.**
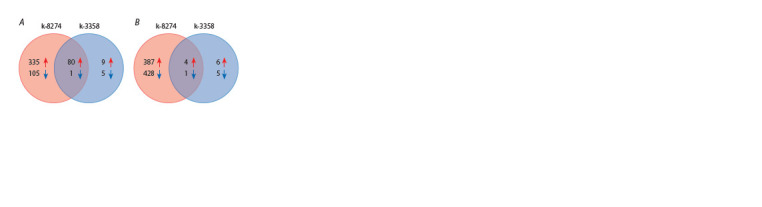
The number of differentially expressed genes in roots of k-8274 and k-3358 under single (А) and double (B) inoculation as compared to uninoculated control.

Inoculation with rhizobia changed the gene expression profiles in roots of both studied genotypes. The response to inoculation was more pronounced in k-8274: 440 unique genes were differentially expressed as compared to control; for k-3358, there were only 14 such genes. Additionally, 81 genes changed their expression similarly in both genotypes (Fig. 1A), and for all but one, the expression level increased. Enrichment analysis for the similarly up-regulated genes showed that these genes were related to the biological processes connected with nodulation and nitrogen fixation, such as biosynthesis of glutamate from proline, 1-aminocyclopropane-1-carboxylate biosynthesis, polyamine transmembrane transport, etc. (Table 1). The genes additionally activated in k-8274 were related to such biological processes as catabolism of polyamines, lipid metabolism, and jasmonic acid and salicylic acid signaling (Table 1).

**Table 1.**
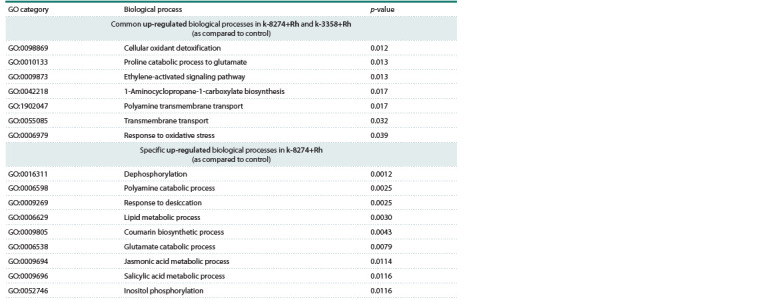
Up-regulated biological processes under single inoculation with rhizobia (Rh) in the studied genotypes

Interestingly, the same genes in k-3358 were not classified as DEGs due to their low expression level both in control and under inoculation with rhizobia. Further, in k-8274, the downregulated genes were related to ion transport, growth and response to fungi, which may indicate a decrease in the AM fungi spread in k-8274 roots. In k-3358, the down-regulated genes were related to the electron transport chain, as well to the response to abscisic acid and water deprivation.

Transcriptomic response to double inoculation (Rh+AM)

The transcriptomic response to double inoculation was different in the studied genotypes (Fig. 1B). Double inoculation of the responsive k-8274 genotype altered the expression of 815 unique genes in the roots, while only 11 unique genes in the non-responsive genotype k-3358 changed their expression level, and 5 genes showed identical expression changes in both genotypes (Fig. 1B). The absolute expression level of the same 815 genes in k-3358 in all tested conditions was comparable to that of k-8274 in control conditions and did not change significantly due to inoculation (the effect similar to that observed under mono-inoculation with rhizobia), reflecting the low responsivity of k-3358 to double inoculation.

Enrichment analysis showed that several up-regulated DEGs in k-8274 under double inoculation (Rh+AM) were related to the same biological processes as in k-8274 under single inoculation (Rh), namely, jasmonic acid signaling, defense response and response to wounding (Table 2). The common down-regulated biological processes included pectin catabolism, inorganic anion transport, and regulation of 1-deoxy-Dxylulose- 5-phosphate synthase activity. Further, many genes specifically up-regulated in k-8274 under double inoculation (Rh+AM) are associated with arbuscular mycorrhiza infection (GO biological processes: oxylipin biosynthetic process, defense response, response to chitin). In turn, the specific down-regulated genes in k-8274 are related to nodulation and assimilation of combined nitrogen (GO biological processes: nodulation, nitrate transmembrane transport) (Table 2). The observed down-regulation of the nodulation-related genes matches the result of phenotypic analysis of the plants (Zhukov et al., 2017): k-8274 plants formed significantly less nodules under double inoculation than in control conditions and under single inoculation with rhizobia.

**Table 2.**
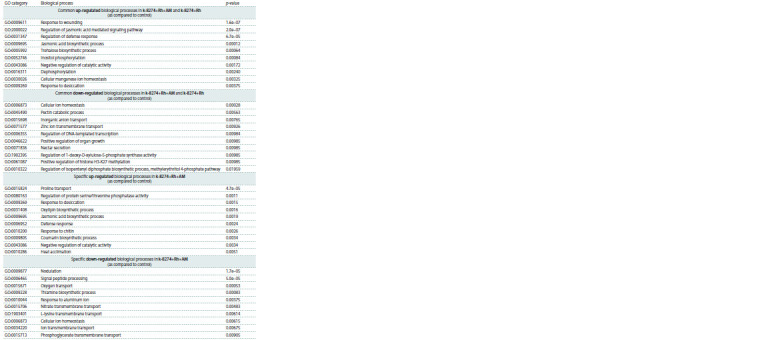
Up- and down-regulated biological processes under double inoculation with rhizobia (Rh) and AM fungi (AM) in the studied genotypes

Comparison of k-8274 and k-3358 under double inoculation

In order to get an insight into the molecular bases of the symbiotic responsivity in pea, we compared the transcriptomes of the ‘responsive’ k-8274 and ‘non-responsive’ k-3358 genotypes treated with Rh+AM. The biological processes up-regulated in k-8274 roots include lignin biosynthesis and cell wall biogenesis, as well as phosphatidylinositol biosynthesis (Table 3). In turn, responses to glucose and fructose, and biosynthesis and metabolism of glutathione and cysteine in k-8274 were down-regulated compared to those in k-3358 (Table 3). The corresponding genes with higher expression level in k-8274 or k-3358 roots are listed in Table S2

**Table 3.**
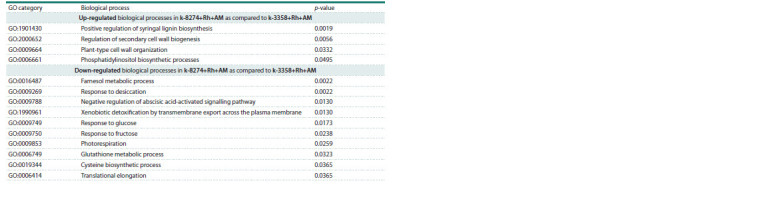
Biological processes that are differentially regulated in roots of k-8274 and k-3358 under double inoculation (Rh+AM)

In our previous study, root transcriptomes of three highly responsive (high-EIBSM) and three low-responsive (low- EIBSM) pea genotypes grown in experimental conditions similar to those of the present experiment (i. e., in pots with soil under combined inoculation with nodule bacteria and AM fungi) were compared, and 90 differentially expressed genes were identified (Afonin et al., 2021). The intersection of the lists of DEGs from the previous and the present experiment revealed 11 genes that were similarly up- or down-regulated in the following comparisons: ‘three high-EIBSM vs three low- EIBSM genotypes’ and ‘k-8274 vs k-3358’ (Table 4). They can be considered as transcriptomic markers of the symbiotic responsivity trait. Interestingly, 5 out of 9 up-regulated genes are related to abscisic acid, jasmonic acid and salicylic acid metabolism and signaling, which points towards the activation of defense reactions in the roots of the high-EIBSM pea plants

**Table 4.**
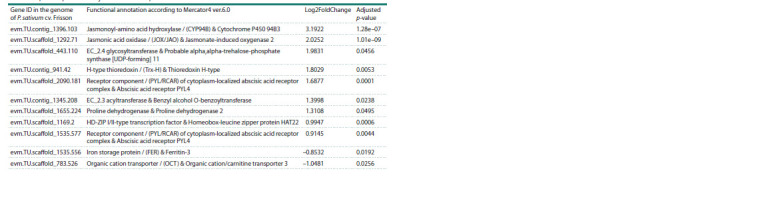
Differentially expressed genes of k-8274 (Rh+AM) as compared to k-3358 (Rh+AM) that overlap in the present study and Afonin et al., 2021

Targeted analysis of marker genes

**Fig. 2.**
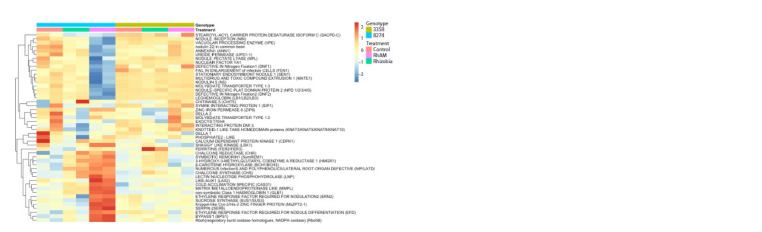
Expression level of selected Sym-genes in roots of k-8274 and k-3358 in control conditions and under single and double inoculation. The heatmap is based on a matrix containing the values of 1–R (R = Pearson correlation coefficient). The raw data were normalized to the size of the dataset, logarithmically transformed, and converted to a z-scale. The lowest gene expression value is represented in blue; the highest value is represented in red.

The expression level of the symbiotic (Sym) genes (i. e., genes, the role of which in AM and/or RN symbiosis was experimentally verified by mutation analysis or RNAi experiments in model legumes) was examined across all RNAseq samples (39 genes, see Table S1). Interestingly, the samples of k-8274 under double inoculation (Rh+AM) strikingly differed from all other samples, showing two clusters of Sym genes, the expression level of which was either higher or lower as compared to other samples (although several genes in the up-regulated cluster had similar expression level in the samples under mono-inoculation with rhizobia) (Fig. 2). The cluster of down-regulated genes contains 15 genes that are normally expressed during nodule development (which again reflects the decreased nodule number in k-8274 plants under double inoculation) and 2 genes (VPE, ANN1) involved in the infection process in both Rh and AM symbioses (Table S1). The cluster of 18 up-regulated genes includes the genes of the common symbiotic signaling pathway (CSSP) (SymREM1, HMGR1). Interestingly, the other genes belonging to CSSP do not demonstrate altered expression, which suggests that the early steps of both AM and nodule development are not disturbed in k-8274.

**Fig. 3.**
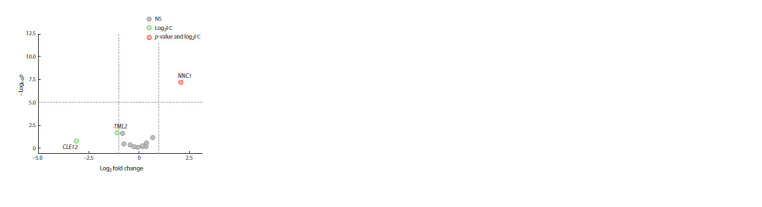
Differential expression of genes related to autoregulation of nodulation in roots of k-8274 under double inoculation as compared to uninoculated control. Log2 fold change (Log2FC) – the binary logarithm of the ratio of a transcript’s expression values in two different conditions; –Log10 p – negative logarithm to base 10 of the p-value. For ease of visualization, the p-values for each gene were log-transformed on the graph; NS – statistically non-significant difference in expression (–Log10 p <5.0, or p < 0.00001). The threshold p-value (p < 0.00001) was chosen taking into account the correction for multiple comparisons. The red color: the NNC1 gene passed both the p-value (–Log10 p > 5.0) and expression level (log2FC > 1.5) significance thresholds; the green color: the CLE12 and TML2 genes have significantly reduced expression levels (log2FC < –1.5), but this reduction is not statistically significant (–Log10 p < 5.0).

The expression level of the genes related to the AON system was also examined. Out of 13 genes tested, the expression level of only one gene, NNC1, was increased in roots of k-8274 under double inoculation (Fig. 3) compared to control. The NNC1 protein in soybean is a suppressor of the master transcriptional regulator NIN (Wang L. et al., 2019), so it seems that the observed down-regulation of Nin in k-8274 under double inoculation (Fig. 2) is also controlled via up-regulation of NNC1, resulting in reduction of nodule formation.

Finally, the expression level of the PsGLP2 gene was tested, which was recently identified as a transcriptional marker of high symbiotic responsivity of k-8274 and its descendent breeding line ‘Triumph’ (Zorin et al., 2023). We detected the trend of up-regulation of PsGLP2 in k-8274 under both monoand double inoculation, as compared to control, although this change in expression level was not statistically significant (Fig. S1). In k-3358, the expression of PsGLP2 was not altered under either single or double inoculation

The suppression of nodulation in k-8274 is condition-dependent

We reanalyzed the raw data of nodule number from Zhukov et al., 2017 by one-way ANOVA and confirmed that AM significantly influenced the nodulation in k-8274 but not k-3358 plants grown in a non-sterile soil (Table 5). However, this result was not reproduced in a new experiment in other experimental conditions (in sterile sand): both k-3358 and k-8274 genotypes demonstrated a decrease in nodulation under combined inoculation (rhizobia + AM fungi) as compared to single inoculation with rhizobia, but the effect was not statistically significant (Table 6).

**Table 5.**
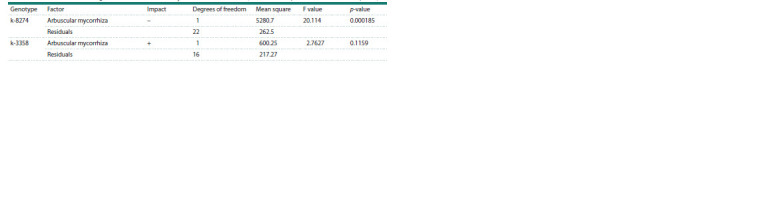
ANOVA results assessing the effect of arbuscular mycorrhiza on nodulation (non-sterile soil, data from Zhukov et al., 2017) Note. Here and in Tables 6 and 7: the ‘Impact’ column indicates an increase (+) or decrease (–) in the mean value due to the estimated factor.

**Table 6.**
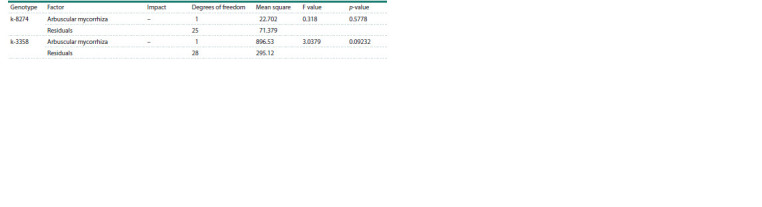
ANOVA results assessing the effect of arbuscular mycorrhiza on nodulation (sterile sand)

In sand, AM treatment decreased the shoot dry weight of the non-responsive genotype k-3358 and did not alter the shoot dry weight of the responsive genotype k-8274, compared to the untreated control (Table 7). One can suggest that in some environments (sterile sand) mycorrhiza begins to harm an ineffective genotype, but not an effective one; thus, the trait of symbiotic responsiveness may manifest itself differently in different experimental conditions.

**Table 7.**
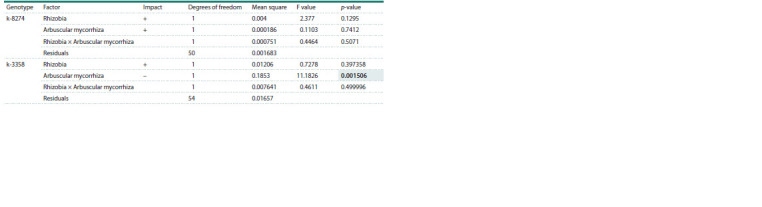
Two-way ANOVA results assessing effects of rhizobia and arbuscular mycorrhiza on shoot weight (sterile sand)

## Discussion

The trait of symbiotic responsivity, or EIBSM (Efficiency of Interaction with Beneficial Soil Microorganisms), is a quantitative trait which is determined as an increase in seed biomass due to inoculation with rhizobia and arbuscular mycorrhizal fungi (Shtark et al., 2012). Apparently, the genetic control of this trait is complex; therefore, preliminary works aimed at molecular genetic characterization of plant genotypes with different symbiotic responsivity are required. In pea, two contrasting genotypes, the ‘responsive’ k-8274 and the ‘nonresponsive’ k-3358, are used as models to study different aspects of manifestation of this trait. Previously, by proteomic analysis of pea seeds, it was shown that the high responsivity to combined inoculation with rhizobia and AM fungi is connected with prolongation of the seed filling period in the responsive’ genotype (Mamontova et al., 2019). The results of the present work expand the description of the responsivity trait: it was demonstrated that the roots of the ‘responsive’ genotype showed a more pronounced reaction to inoculation at the transcriptomic level than the roots of the ‘non-responsive’ one, and that the reaction of the ‘responsive’ genotype to combined inoculation (rhizobia + AM fungi) involved downregulation of the nodule-related genes, which is in line with the suppression of nodulation shown in the earlier experiments (Zhukov et al., 2017).

Although the bulk transcriptome analysis of the entire root system does not allow accurate assessment of gene expression (since the development of nodules and/or arbuscular mycorrhiza may be regulated differently in different zones of the roots), the effect of inoculation was clearly visible in the ‘responsive’ genotype in contrast to the ‘non-responsive’ one. The list of genes with an elevated expression in the roots of k-8274 (as compared to k-3358) under double inoculation includes the genes encoding Lipid Transfer Protein (LTP) family member, putative SOUL heme-binding protein, MYB-like transcription factor, expansin, and metallothionein protein (Table S2). Some of these genes are directly linked to nodulation and/or mycorrhization: one of the members of the LTP family in M. truncatula known as Nodulin 5 is required for the successful symbiosis with Sinorhizobium meliloti (Pii et al., 2009); expansins play a role in arbuscular mycorrhiza formation (Mohanty et al., 2018); a member of metallothionein family is involved in rhizobial infection and nodulation in Phaseolus vulgaris (Fonseca-García et al., 2022).

Interestingly, several DEGs obtained in this comparison were also found within the previously published list of transcriptomic signatures characteristic of roots of symbiotically responsive pea genotypes grown in a non-sterile soil (Afonin et al., 2021). It is important to note that the experiment of Afonin and colleagues did not include either k-8274 or k-3358, thus, the resulting intersection of the gene lists from Afonin et al., 2021 and the present study may be considered as a list of reliable transcriptomic markers of the EIBSM trait (Table 4). These markers include the up-regulated genes that encode the enzymes jasmonoyl-amino acid hydroxylase and jasmonic acid oxidase, two different genes encoding the abscisic acid receptor PYL4 (which is also involved in jasmonic acid signaling; Lackman et al., 2011), and a benzyl alcohol O-benzoyltransferase, which is involved in biosynthesis of salicylic acid (Kotera et al., 2023). Salicylic acid and jasmonates play a key role in plant defense and have a strong influence on plants metabolism (Jeyasri et al., 2023; Monte, 2023); thus, the manifestation of the EIBSM trait may be based on the fine-tuning of defense reactions accompanied by metabolic changes. Moreover, it can be hypothesized that interaction with beneficial soil microorganisms may have a positive effect on systemic resistance of k-8274 plants, and this aspect of the EIBSM trait manifests itself in non-sterile soil but not in sterile sand.

In k-8274, the double inoculation with nodule bacteria and AM fungi led to down-regulation of the genes normally expressed in nodules, which corresponds to the previously described phenotype of plants (suppression of nodulation; Zhukov et al., 2017) and perhaps reflects the optimization of the nitrogen nutrition of the plants. Interestingly, the early symbiotic genes were not suppressed under double inoculation, indicating that CSSP, which is common for both AM and RN symbioses, functioned normally in these conditions. This means that the down-regulation of RN symbiosis takes place after the common signaling pathway, apparently, in order not to block the development of both symbioses together. Probably, a similar block of the symbiosis development occurs when pea interacts with non-specific rhizobia (this is the case for Afghanistan peas, a group of varieties which can form nodules only with a low number of specific strains, as opposed to European peas, which are nodulated by a broad spectrum of strains (Lie, 1984; Firmin et al., 1993)). In this case, the phenotypic analysis suggests that the early steps of symbiosis (encoded by CSSP genes) proceed normally, but the penetration of rhizobia into the root hairs is blocked due to absence of the signal transduction mediated by the receptor kinase LykX (=Sym2) (Sulima et al., 2017, 2019). It would be interesting to assess the expression level of the PsNNC1 gene, which was up-regulated in k-8274 under double inoculation, in Afghanistan peas interacting with non-specific rhizobia, in order to check whether it participates in signal transduction during specific and non-specific interactions with rhizobia.

The nodulation suppression detected in k-8274 under double inoculation is accompanied by a decrease in the expression level of the PsCLE12 and PsTML2 genes (Log2FC < –1.0, although this decrease is not statistically significant, see Fig. 3). Orthologs of these genes in M. truncatula act as negative regulators of nodule development and are parts of the AON (autoregulation of nodulation) system (Gautrat et al., 2019). This observation is consistent with our previous suggestion regarding the possible connection between the AON system and symbiosis efficiency (Zhukov et al., 2021b).

The phenomenon of nodulation suppression was observed in non-sterile soil (Zhukov et al., 2017), where plenty of microorganisms occur, whereas in sterile sand, the decrease in nodule number under double inoculation (Rh+AM) was visible but non-significant for both studied genotypes. Also, in sand, the inoculation with AM fungi had a negative effect on the ‘non-responsive’ genotype k-3358 and was neutral to the ‘responsive’ genotype k-8274. One can conclude that the responsivity trait may be dependent on several environmental factors such as temperature, humidity, the presence of indigenous microorganisms in the growth substrate, etc. Therefore, large-scale experiments are required to estimate the percentage of genotype (G) effect on the manifestation of the symbiotic responsivity trait in comparison to environment (E) and genotype-environment (G × E) interaction.

Recently we showed that the plant’s habitus plays a role in manifestation of the responsivity trait: pea genotypes bearing a natural mutation le (p.A229T) in the Le gene encoding gibberellin 3-beta-dioxygenase (Martin et al., 1997) have shortened internodes, lower biomass and are more responsive to double inoculation (Rh+AM) than wild-type genotypes (Zhukov et al., 2021a). One of the explanations for this phenomenon was that smaller plants could react more quickly to change in the nitrogen/phosphorous content in the roots and inhibit formation of new symbiotic structures, since this reaction is mediated by long-distance signaling involving CLE and CEP peptides (Okamoto et al., 2016). Indeed, we showed that pea genotypes with long stems had more AM in their roots than the genotypes with short stems (Zhukov et al., 2021a), and in the present study, we found that the down-regulation of nodulerelated genes in non-sterile soil is characteristic of k-8274, which has the le phenotype, as opposed to k-3358 with the Le phenotype. Thus, the pleiotropic effect of the le mutation may also include influence on the plants’ symbiotic responsivity, probably due to quicker signaling, which leads to suppression of formation of excessive symbiotic structures; however, further experiments are required to prove this statement

## Conclusion

Due to the development of pea genomics, genome- and transcriptome- wide analyses became available, making it possible to uncover the molecular bases of the traits of interest, including the symbiotic responsivity trait. Here, we described the transcriptomic signatures characteristic of roots of the symbiotically responsive k-8274 genotype. The biological processes associated with the functions of the identified genes include lignin biosynthesis, cell wall biogenesis, and biosynthesis of phosphatidylinositol. Also, the ‘responsive’ genotype k-8274 demonstrated the pronounced change in the gene expression profiles in roots, as opposite to the ‘non-responsive’ genotype k-3358, which reflects the observed differences in the effect of inoculation with symbiotic microorganisms. Further work should be devoted to the search for specific genes that affect EIBSM, which will form the basis for marker-assisted selection of new pea cultivars with high effectiveness of interaction with nodule bacteria and arbuscular mycorrhizal fungi.

## Conflict of interest

The authors declare no conflict of interest.
